# Association of Proliferative Indices With Various Grades of Breast Carcinoma

**DOI:** 10.7759/cureus.41865

**Published:** 2023-07-14

**Authors:** Seema Awasthi, Pooja Gupta, Ankita Mittal

**Affiliations:** 1 Department of Pathology, Teerthanker Mahaveer Medical College, Moradabad, IND

**Keywords:** indices, proliferative, carcinoma, ki-67, agnor

## Abstract

Aims and objectives: Various grades of breast carcinoma and proliferative indices used as nuclear protein Ki-67 and argyrophilic nucleolar organizer regions (AgNOR) are being compared to each other.

Materials and method: In this observational cross-sectional investigation, 42 breast biopsies from questionable breast areas were collected and preserved in formalin and paraffin before the tissue blocks were made. A thorough medical history regarding the breast tumor and thorough physical examination results were recorded. Two sections were produced, one stained with an immunohistochemical marker called Ki-67 and the other with a unique stain called AgNOR.

Results: Grade I in Nottingham was found to be highest in subjects with Ki-67 1%, grade II in subjects with Ki-67 1-10%, and grade III in subjects with Ki-67>10%. Therefore, a higher Ki-67 score and a higher Nottingham grade were more closely associated. The mean AgNOR score was determined to be highest in Nottingham grade III and lowest in Nottingham grade I. In contrast to grade I and grade II of carcinoma (CA) breast, where there was no statistically significant association between Ki-67 and AgNOR, grade III of CA breast showed a statistically significant link between Ki-67 and AgNOR.

Conclusion: Proliferation has been identified as a distinctive feature of cancer and as a key factor in the prognosis of the disease.

## Introduction

Breast cancer is a condition with a wide range of symptoms, including biological characteristics and clinical behaviors [1,2]. Breast cancers are clonal proliferations that develop from cells with a variety of genetic abnormalities. Both inherited susceptibility genes and hormonal exposures contribute to these genetic abnormalities. An estimated 12% of breast cancer cases are thought to be caused by the inheritance of known susceptibility genes. The breast cancer susceptibility gene 1 (BRCA1) and BRCA2 genes are mutated in around 3% of breast cancer cases. Exposure to hormones, gender, menarche and menopause age, reproductive history, and nursing are important risk factors for sporadic breast cancer. Another red flag is having a close relative who has breast cancer [3].

Breast tumors with comparable histological characteristics might present differently clinically, have different disease aggressiveness, and respond differently to a treatment. Breast cancer and other malignancies have been reclassified at the molecular level as a result of systematic analyses of gene expression patterns and their associations with certain phenotypic variation factors. Our understanding of the phenotypic variability exhibited in cancers is being improved by this change [4,5].

It is significant to note that there are at least three diseases, ductal carcinoma in situ (DCIS), invasive ductal carcinoma (IDC), and invasive lobular carcinoma (ILC), that may be separated from one another both molecularly and clinically, and that breast cancer may potentially develop from a range of distinct progenitor cells. It is now possible to comprehend the molecular profile of cancer because of improvements in technology, particularly the microarray [6].

Argyrophillic nucleolar organizer regions (AgNORs), a particular class of molecular cancer markers, are referred to as NORs. NORs are deoxyribonucleic acid (DNA) loops that can be discovered in a cell's nucleus, specifically on the acrocentric chromosomes [7,8]. Nuclear envelope regions are the name given to these loops. Polymerase C23 and B23 are two instances of the so-called argyrophilic proteins, which are known for their affinity for silver. These argyrophilic-associated proteins can be identified using a straightforward silver staining approach [9]. Quiescent and actively dividing cells (those in the G1, S, or M phase) do not contain the Ki-67 antigen. The concentrations of Ki-67 are lowest during the G1 and S phases and are largest at the start of the mitotic phase. Ki-67 levels drastically decrease as mitosis progresses and the cell gets ready to divide [10].

The goal of the current study was to develop a new technique for precise and accurate tumor grading that could be utilized in conjunction with routine histopathology findings to produce more accurate prognostic data. This was done by employing the Ki-67 and AgNOR stains to assess the proliferative activity of cancer cells.

## Materials and methods

Forty-two breast excisional biopsies were used in this observational cross-sectional investigation. The samples were taken from questionable breast locations, formalin-fixed, and paraffin-embedded tissue blocks were created. The study included all breast cancer samples that met the inclusion and exclusion criteria. A thorough medical history regarding the breast tumor and thorough physical examination results were recorded. They play a crucial role in diagnosis, treatment planning, prognosis determination, monitoring, and research, ultimately contributing to better patient care and outcomes. AgNOR was used as a particular stain on one of the two prepared sections, while Ki-67 was used as an immunohistochemistry marker on the other.

Study population

Following clearance from the ethics committee of the Teerthanker Mahaveer University (Approval No. TMU/IEC/20-21/099), all breast biopsy samples identified at the Department of Pathology as having breast cancer and meeting the inclusion and exclusion criteria were included in the study. Breast biopsies identified histopathologically as breast cancer were included in the study. The study eliminated individuals who refused to provide informed consent, autolyzed specimens, inadequate breast biopsies, and other cancers that metastasized to the breast.

Assessment of immunohistochemistry (IHC) staining

The percentage of expression of Ki-67 by tumor cells was determined based on the tumor cells that were stained positively for nuclear staining. Yamashita et al.'s grading standards were used to determine the results [11]. Total tumor cell counts ranged from 100 to 500, and of those 500 cells that displayed the highest immunoreactivity and were expressed as a percentage, the number of positive Ki-67 cells was counted and multiplied by 100. Grading was done as 0 = None, 1 = <1%, 2 = 1-10%, 3 = 10-50%, 4 = >50%. Ki-67 expression was regarded to be present in tumors with a score of 2 or higher.

The light microscope equipped with a silver staining technique was used. In this nucleolus and within the nucleoplasm, AgNOR is seen as blackish or brown spots on a light-yellow background. The number of AgNORs found inside the nuclei of 100 cancer cells was counted using a 100X objective. The results were then reported as the mean plus or minus the standard deviation after computing the mean numbers of NORs per nucleus. The result was correlated with histological grading of tumors and appropriate statistical analysis was performed.

Statistical analysis

Upon loading, the Excel data was examined using Statistical Package for Social Sciences (SPSS) version 25.0 (IBM Corp. Armonk, NY). While qualitative (categorical variables) data were presented as frequency and percentage, quantitative (numerical variables) data were presented as mean and standard deviation. The chi-square test was used to examine the frequency differences between the two groups, and the student t-test was used to compare the means of the two groups. Correlation between the variables was done using Pearson's correlation coefficient test where the p-value was statistically significant if p≤0.05.

## Results

The ages of study participants ranged from 22 to 80, with 22 being the youngest and 80 being the oldest. The group's average age was 50.88±14.59 years. Of the participants, 38%, 57.1%, and 2.4%, respectively, reported having left, right, and recurring breast lumps. Of the research participants, 90.5%, 4.76%, 2.38%, and 2.38%, respectively, had invasive ductal carcinoma (NST), carcinoma with medullary characteristics, invasive lobular carcinoma, and low-grade ductal cancer. Of the subjects, 16.7%, 23.8%, and 59.5%, respectively, had Nottingham grades I, II, and III (Table 1).

**Table 1:** Baseline characteristics of the study population

Variables	Characteristics	Number	Percentage
Side of breast affected	Left breast lump	17	40.5
	Right breast lump	24	57.1
	Recurrent breast lump	1	2.4
Diagnosis	Invasive ductal carcinoma (NST)	38	90.5
	Carcinoma with medullary features	2	4.8
	Invasive lobular carcinoma	1	2.4
	Low-grade ductal carcinoma	1	2.4
Grade	Grade I	7	16.7
	Grade II	10	23.8
	Grade III	25	59.5
Age group (in years)	Mean	50.88	
	Standard deviation	14.59	
	Range	22-80	

Grade I in Nottingham was found to be highest in subjects with Ki-67 1%, grade II in subjects with Ki-67 1-10%, and grade III in subjects with Ki-67>10%. Therefore, a higher Ki-67 score and a higher Nottingham grade were more closely associated. The mean Ki67 score was highest in Nottingham grade III and lowest in Nottingham grade I, a difference that was statistically significant (Table 2).

**Table 2:** Association of Nottingham grade with Ki-67 score

Nottingham grade		Ki-67 score					
		<1%	1-10%	10-50%	>50%	Mean	Standard deviation
Grade I	N	5	1	1	0	5.84	12.93
Grade II	N	2	4	2	2	22.76	25.53
Grade III	N	2	7	11	5	34.10	26.91
p-value		0.022*				0.002*	

Grade I in Nottingham was found to be the highest in subjects with an AgNOR score of 4-6, grade II in subjects with an AgNOR score of 2-4, and grade III in subjects with an AgNOR score of 4-6. AgNOR score was used to compare Nottingham grade distribution, and a statistically insignificant difference (p>0.05) was discovered. Although there was no noticeable difference, the mean AgNOR score was highest in Nottingham grade III and lowest in Nottingham grade I (Table 3).

**Table 3:** Association of Nottingham grade with AgNOR score AgNOR: argyrophilic nucleolar organizer regions

Nottingham grade		AgNOR score				
		2-4	4-6	6-8	Mean	Standard deviation
Grade I	N	3	4	0	4.07	0.97
Grade II	N	6	1	3	4.67	1.97
Grade III	N	10	13	2	4.71	1.35
p-value		0.090			0.31	

In contrast to grade I and grade II of carcinoma (CA) breast, where there was no significant link between Ki-67 and AgNOR, grade III of CA breast did have a strong relationship between Ki-67 and AgNOR (Table 4).

**Table 4:** Correlation of AgNOR with Ki-67 AgNOR: argyrophilic nucleolar organizer regions

Variable	Stage	Test and p-value	AgNOR		
			Stage I	Stage II	Stage III
Ki-67	Stage I	Pearson's correlation	-0.386		
		p-value	0.392		
	Stage II	Pearson's correlation		0.095	
		p-value		0.794	
	Stage III	Pearson's correlation			0.466
		p-value			0.019

**Figure 1:**
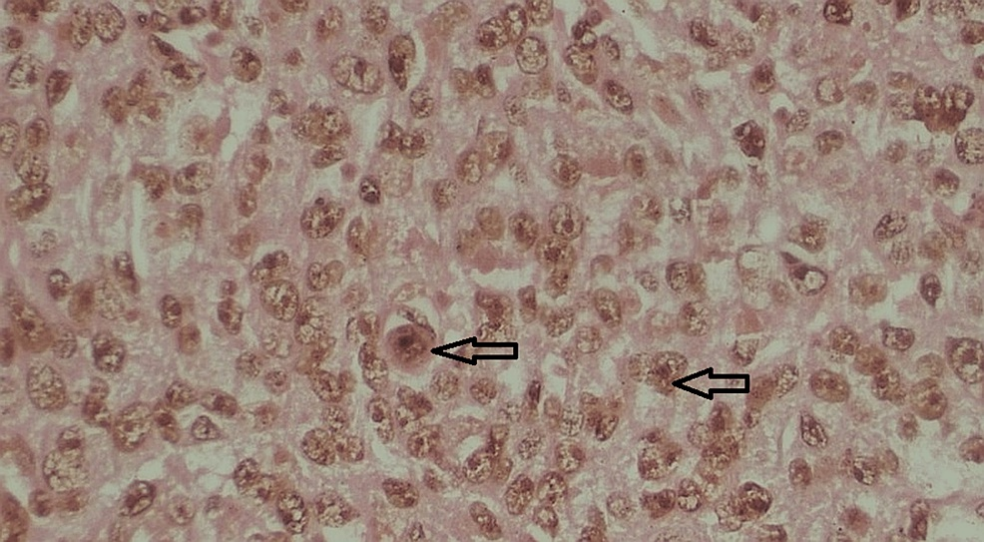
Grade 3 invasive ductal carcinoma showing mean AgNOR count of range 6-8 AgNOR: argyrophilic nucleolar organizer regions

**Figure 2:**
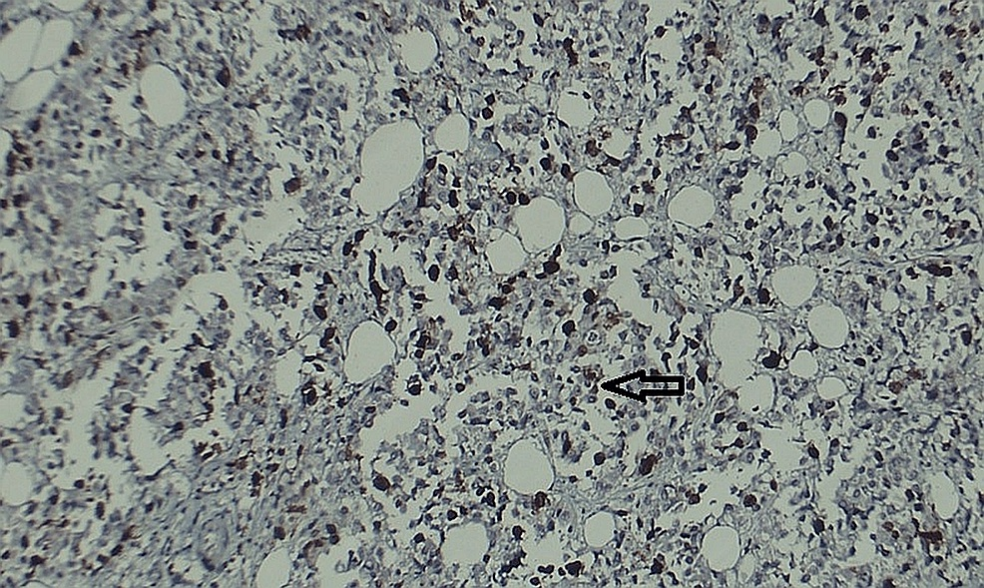
Grade 3 invasive ductal carcinoma showing score 4 nuclear staining for Ki-67 marker

Histopathological staining of the samples revealed the markers present as shown in Figure 1 and Figure 2.

## Discussion

The most prevalent kind of cancer in women and the leading factor in cancer-related mortality in females is breast cancer. The World Health Organization (WHO) estimates that cancer causes 7.6 million deaths worldwide each year, with breast cancer accounting for 502,000 of those fatalities. The deployment of intensive multimodal treatment, which includes targeted therapy, and improvements in early illness detection has resulted in a significant decrease in the death rate during the last 20 years [12]. The most promising method for identifying nuclear proteins involved in DNA replication is immunohistochemistry (IHC) analysis. These proteins, like Ki-67, a labile non-histone nuclear protein, are produced by cells that are in the proliferative phase of the cell cycle. It is expressed from the G1 phase through the M phase of the cell cycle and is undetectable in the G0 phase. Consequently, Ki-67 is a very helpful marker [13].

The minimum and maximum ages for the study participants were 22 and 80 years old, respectively, and the mean age was 50.88±14.59 years. It was consistent with research by Ansari et al., in which the mean age was 48.2 years, Setyawati et al., who found the mean age to be 52 years, and Cheng et al., who discovered the mean age to be 48.5 years [14-16]. Consequently, breast cancer and aging are connected. In this study, 90.5%, 4.76%, 2.38%, and 2.38% of the study individuals, respectively, had invasive ductal carcinoma (NST), carcinoma with medullary characteristics, invasive lobular carcinoma, and low-grade ductal cancer.

Out of 70 instances in the study by Karangdan et al., 54 cases (90%) were of the IDC, NST subtype, and similar findings were noted in the study by Mittal et al., in which 66% of the cases were of the IDC, NST subtype and matched the findings of the present study [17,12]. ILC accounted for 2 occurrences (1.7%), while medullary carcinoma and lymphoma each had one case (0.8%). A total of 14 cases of lobular carcinoma, 3 cases of mucinous carcinoma, 2 cases of medullary carcinoma, and 1 case each of secretory carcinoma, papillary carcinoma, and metaplastic carcinoma were found in the study by Ansari et al. [14].

In this investigation, Nottingham grades I, II, and III were present in 16.7%, 23.8%, and 59.5% of the patients, respectively. Similar results were reported by Setyawati et al. [15]. All molecular subtypes were predominantly of grade III, which suggests that the identification of breast cancer was delayed in the Indonesian investigation. In their study, Ansari et al. found that the majority of the carcinomas (56.8%) belonged to grade 2, followed by grades 1 and 3 (28.7% and 14.5%, respectively) [14]. According to Shukla et al., 51% of the cases fell into the grade II category [18]. In their investigation, Mittal et al. found that the majority of cases had histological grades I and II [12]. Different inclusion and exclusion criteria could be the cause of the grading discrepancy.

Grade I in Nottingham was determined to be at its highest in subjects with Ki-67 1%, grade II in those with Ki-67 1-10%, and grade III in those with Ki 67>10%. Therefore, a higher Ki-67 score and a higher Nottingham grade were more closely associated. According to the Ki-67 using the chi-square test, there was a statistically significant difference (p<0.05) between the Nottingham grade distributions. With a statistically significant difference (p<0.05), the mean Ki-67 score was found to be at its highest in Nottingham grade III and at its lowest in Nottingham grade I.

A positive result for Ki-67 was observed in 12 cases, according to a study by Sharma et al., with a percentage of positivity ranging from 2.33 to 5.516 in grade II tumors and from 9.67 to 7.671 in grade III tumors [19]. They discovered a link between Ki-67 and the grade of the tumor, which is compatible with the results of the current analysis. This is consistent with the findings of studies by Wojnar A et al. and Azambuja et al., which found that grade III tumors had a significantly larger mean number of Ki-67 positive cells than grade II and grade I tumors [20,21].

In the current study, 50% of the participants had a Ki-67 percentage value more than 10. Sharma et al. noted that the proportion of cells that were Ki-67 positive was 30%, despite the fact that other studies have found that it can range from 49% to 53.6% [19]. There were 45.2%, 42.9%, and 11.9% of the study subjects who had an AgNOR score of 2-4, 4-6, and 6-8, respectively. Grade I in Nottingham was found to be the highest in subjects with an AgNOR score of 4-6, grade II in subjects with an AgNOR score of 2-4, and grade III in subjects with an AgNOR score of 4-6. AgNOR score was used to compare Nottingham grade distribution, and a statistically insignificant difference (p>0.05) was discovered. Although there was no statistically significant difference observed (p>0.05), it was discovered that Nottingham grade III had the highest mean AgNOR score, and Nottingham grade I had the lowest mean AgNOR score. However, it was not determined that this difference was statistically significant.

Sharma et al.'s findings indicate that the average number of AgNORs discovered throughout their analysis ranged from 2.42 to 6.68 [19]. In comparison to grade II cancers (3.39), the AgNOR count was on average considerably greater in grade III tumors (4.28). The grade III population had significantly higher mean AgNOR counts than the grade II group, according to research by Dube et al. [22].

In our study, there was no statistically significant link between the Ki-67 and AgNOR results together with regard to CA Breast grades I and II, however, there was a correlation between the Ki-67 and AgNOR scores with regard to CA Breast grades I and III. Although both the measures (score and count) rose with a higher tumor grade, Sharma et al. stated that they could not discover a significant link (p = 0.606) [19].

The study's limitation is the tiny sample size. According to the study that has been done thus far, there is a difference of up to 39% between the molecular categorization offered by IHC and that provided by gene expression. Additionally, there is an urgent need to look into the relationship between molecular subtypes and risk factors in a huge population that is dispersed across the country and in several places.

## Conclusions

Proliferation assessment is one of the key variables in determining a patient's course of treatment for breast cancer, in addition to the traditional histological prognostic markers. Proliferation has been identified as a distinctive feature of cancer and plays a significant role in how the disease will progress. It has been determined that Ki-67 positivity and AgNOR count rise with increasing breast cancer grade and may be linked to a bad prognosis. Consequently, it has an expected predictive importance.
